# Supplementary material for: Exploiting the role of nanoparticle shape in enhancing hydrogel adhesive and mechanical properties
Source: Nat Commun. 2020 Mar 17;11:1420. doi: 10.1038/s41467-020-15206-y (PMC7078206; doi:10.1038/s41467-020-15206-y)
Supplement: Supplementary file 1 — Supplementary Information [file 41467_2020_15206_MOESM1_ESM.pdf]

Supplementary Information for

Exploiting the Role of Nanoparticle Shape in Enhancing Hydrogel Adhesive and Mechanical Properties

Maria C. Arno*, Maria Inam, Andrew C. Weems, Zehua Li, Abbie L.A. Binch, Christopher I. Platt, Stephen Richardson, Judith A. Hoyland, Andrew P. Dove* and Rachel K. O'Reilly*

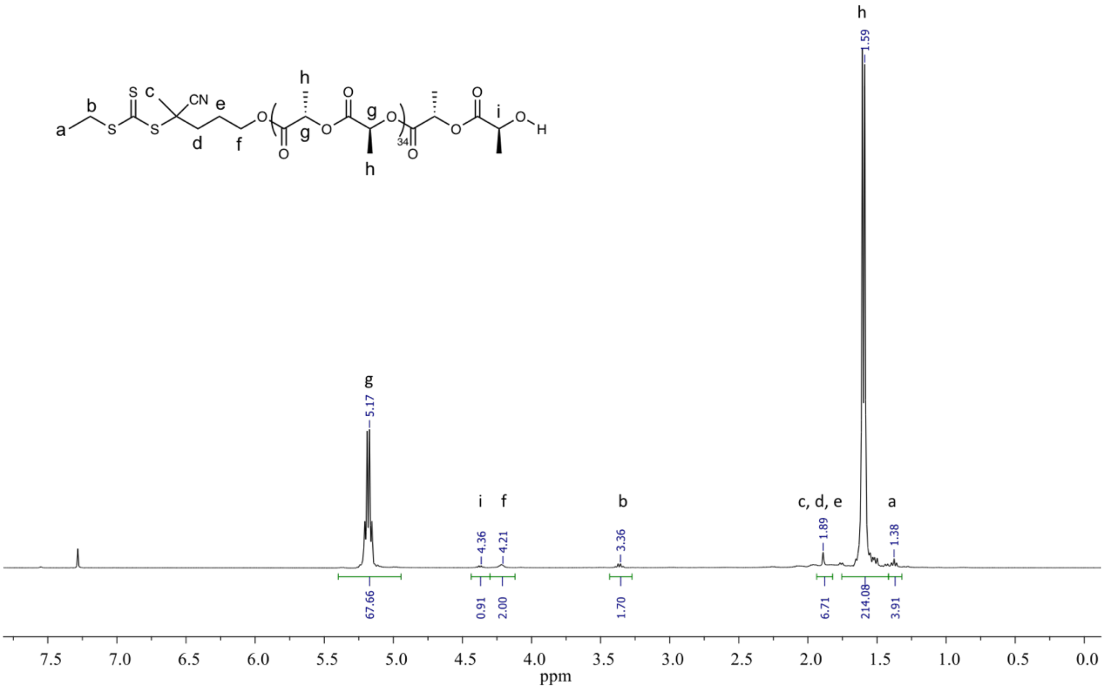

Supplementary Figure 1. ^{1}H NMR spectrum (400 MHz, $CDCl_3$) of $PLLA_{35}$.

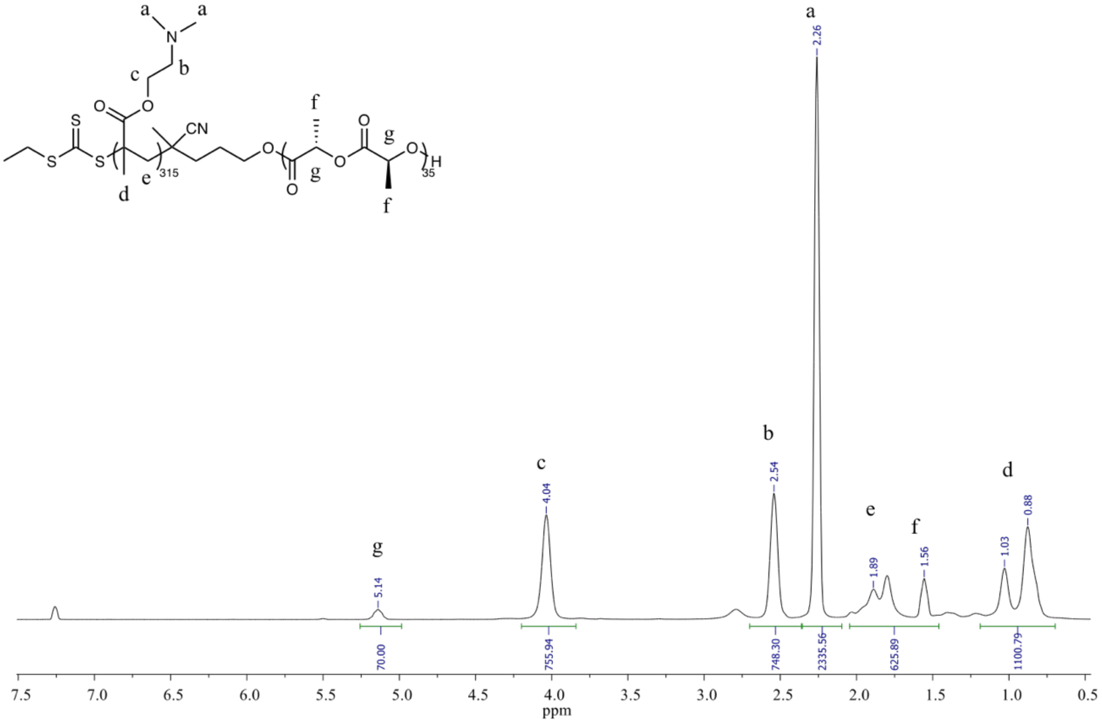

Supplementary Figure 2. ^{1}H NMR spectrum (400 MHz, $CDCl_3$) of $PLLA_{35}$-*b*-$PDMAEMA_{315}$.

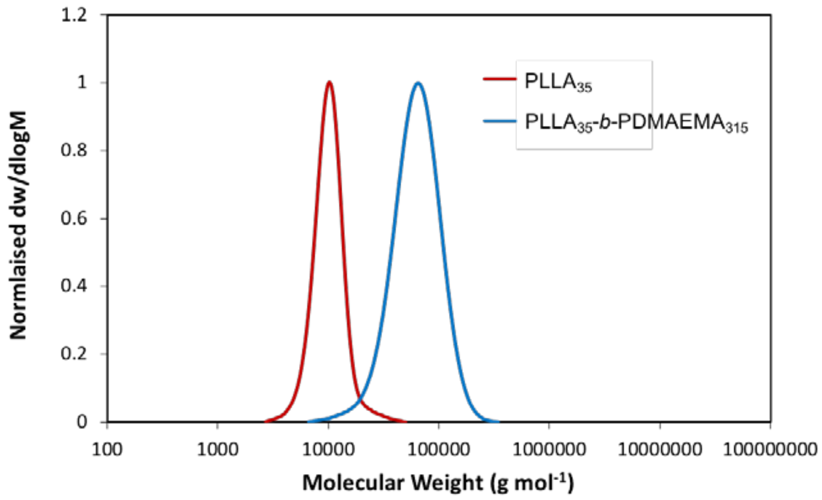

Supplementary Figure 3. Size exclusion chromatograms of $PLLA_{35}$ and $PLLA_{35}$-*b*-$PDMAEMA_{315}$ (RI detection against PMMA standard, DMF).

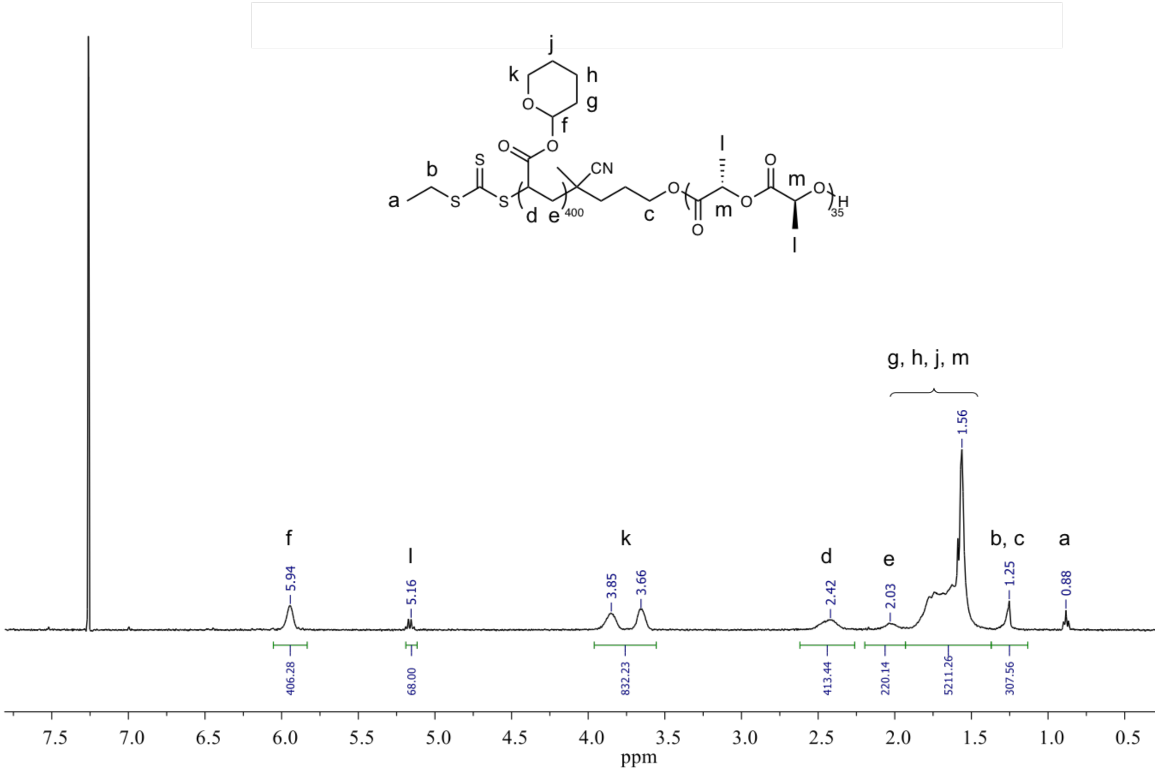

Supplementary Figure 4. ^{1}H NMR spectrum (400 MHz, $CDCl_3$) of $PLLA_{35}$-*b*-$PTHPA_{400}$.

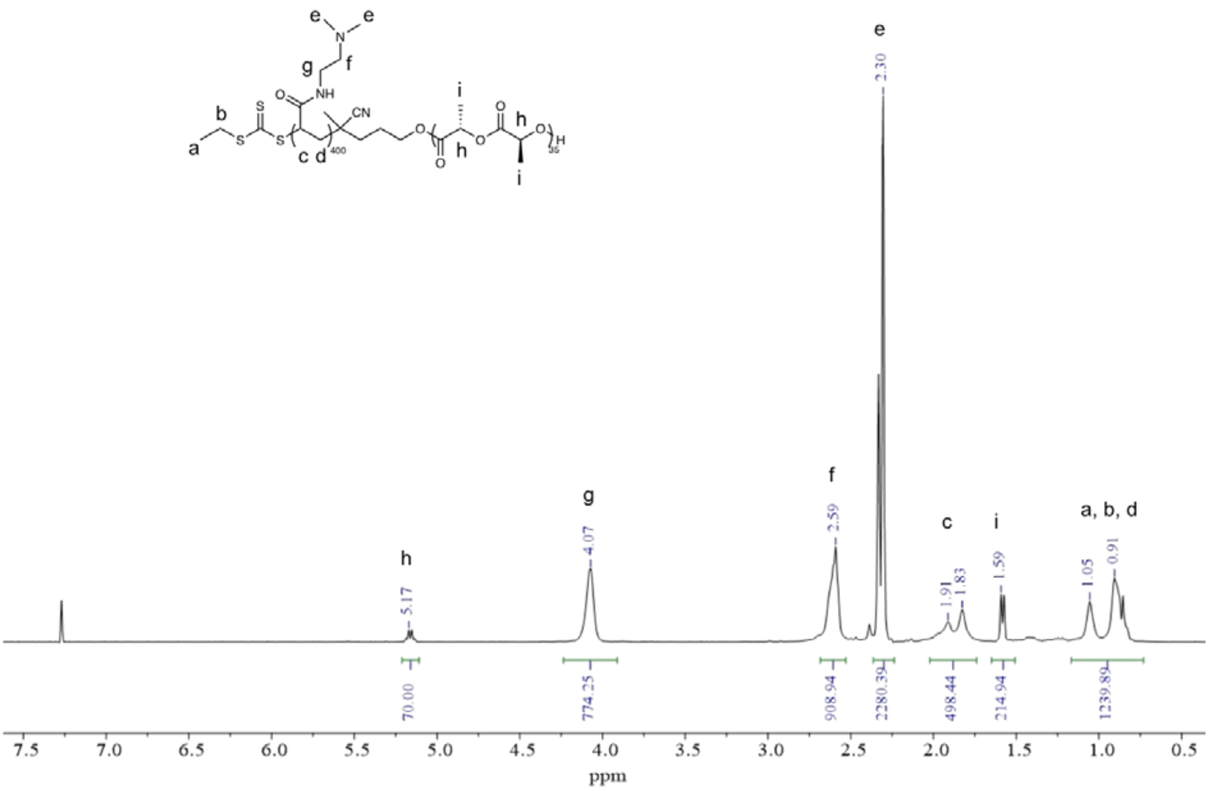

Supplementary Figure 5. ^{1}H NMR spectrum (400 MHz, $CDCl_3$) of PLLA$_{35}$-*b*-PDMAEAm$_{400}$.

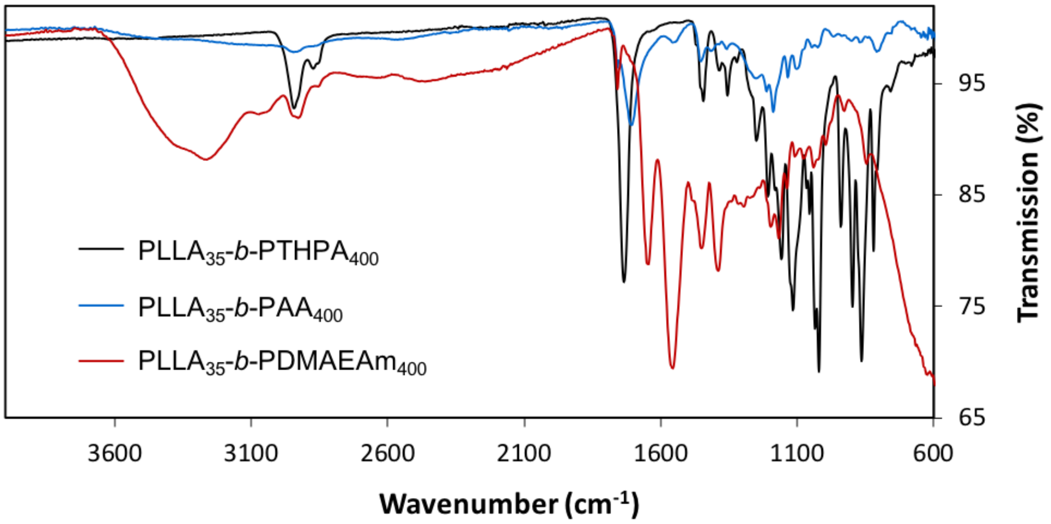

Supplementary Figure 6. FTIR spectra of PLLA$_{35}$-*b*-PTHPA$_{400}$, PLLA$_{35}$-*b*-PAA$_{400}$, and PLLA$_{35}$-*b*-PDMAEAm$_{400}$.

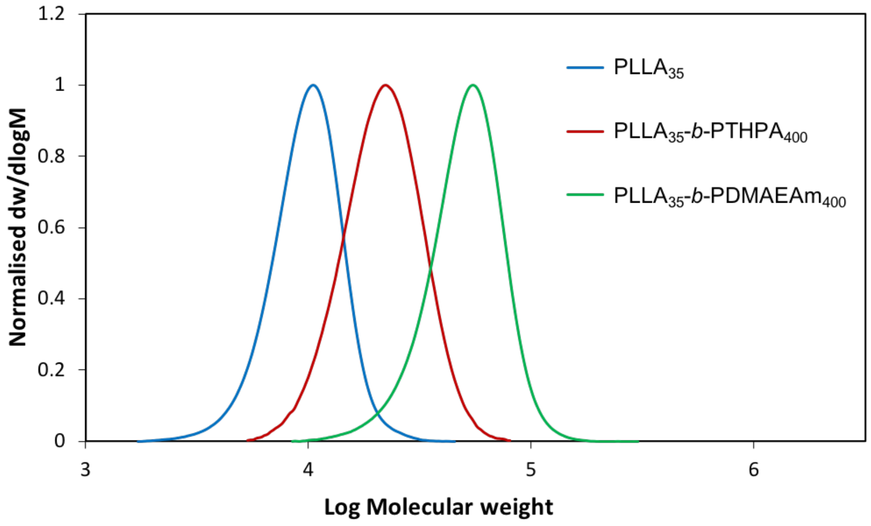

Supplementary Figure 7. Size exclusion chromatograms of $PLLA_{35}$, $PLLA_{35}$-*b*-$PTHPA_{400}$, and $PLLA_{35}$-*b*- $PDMAEAm_{400}$ (RI detection against PMMA standard, DMF).

Supplementary Table 1. Zeta potentials of polylactide-based nanostructures measured in water at pH 2.

	Zeta Potential (mV)
$PLLA_{35}$-*b*-$PDMAEMA_{315}$ small platelets	+ 30
$PLLA_{35}$-*b*-$PDMAEMA_{315}$ medium platelets	+ 31
$PLLA_{35}$-*b*-$PDMAEMA_{315}$ large platelets	+ 29
Quaternized $PLLA_{35}$-*b*-$PDMAEMA_{315}$ platelets	+ 33
Zwitterionic $PLLA_{35}$-*b*-$PDMAEMA_{315}$ platelets	- 4[a]
Anionic $PLLA_{35}$-*b*-PAA_{400} platelets	-30
$PLLA_{35}$-*b*-$PDMAEMA_{315}$ spherical micelles	+ 28.6
$PLLA_{35}$-*b*- $PDMAEAm_{300}$ cylindrical micelles	+ 30

[a]Zeta potential measured at pH 10.

Supplementary Table 2. Dimensions of $PLLA_{35}$-based nanoparticles. Length and width of the nanoparticles were calculated from TEM images using ImageJ analysis software; the height of platelets was obtained from AFM images and the area of spheres was calculated from the diameter obtained by TEM (Table S3).

	**Length, nm (average ± standard deviation)**	**Width, nm (average ± standard deviation)**	**Height, nm (average ± standard deviation)**	**Area, cm^2 (average ± standard deviation)**
**Small platelets**	372 ± 83	223 ± 41	11.0 ± 1.7	$9.3 \times 10^{-10} \pm 8.7 \times 10^{-10}$
**Medium platelets**	893 ± 100	528 ± 60	9.4 ± 1.1	$4.9 \times 10^{-9} \pm 5.0 \times 10^{-8}$
**Large platelets**	1700 ± 169	993 ± 91	10.4 ± 0.8	$1.7 \times 10^{-8} \pm 6.2 \times 10^{-7}$
**Spheres**	n/a	n/a	n/a	$7.1 \times 10^{-10} \pm 3.4 \times 10^{-10}$
**Cylinders**	300 ± 52	10 ± 0.9	n/a	$9.6 \times 10^{-11} \pm 1.6 \times 10^{-11}$

Supplementary Table 3. Distribution of $PLLA_{35}$-*b*-$PDMAEMA_{315}$ spherical and $PLLA_{35}$-*b*-$PDMAEAm_{400}$ cylindrical micelle sizes (average from 300 particles ± standard deviation).

**Spherical micelles**	D_h = 149 nm[a], PDI = 0.245[a] ; 150 ± 37 nm[b]
**Cylindrical micelles**	Length = 300 ± 52 nm[b], Width = 10 ± 1 nm[b]

[a]Obtained by Dinamic light scattering (DLS); [b]Obtained from TEM images using ImageJ analysis software.

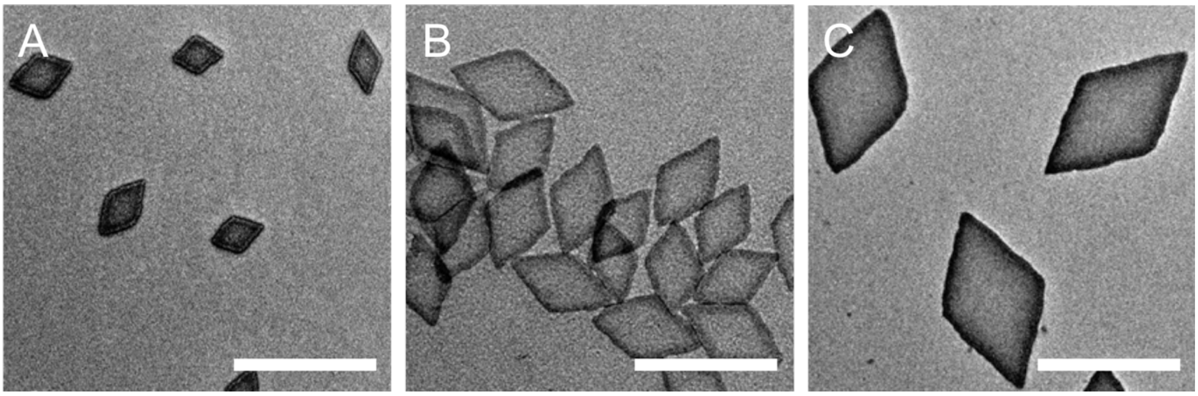

Supplementary Figure 8. Characterisation of PLLA$_{35}$-*b*-PDMAEMA$_{315}$ platelets. TEM micrographs of small (A), medium (B), and large (C) platelets. Scale bar = 2000 nm.

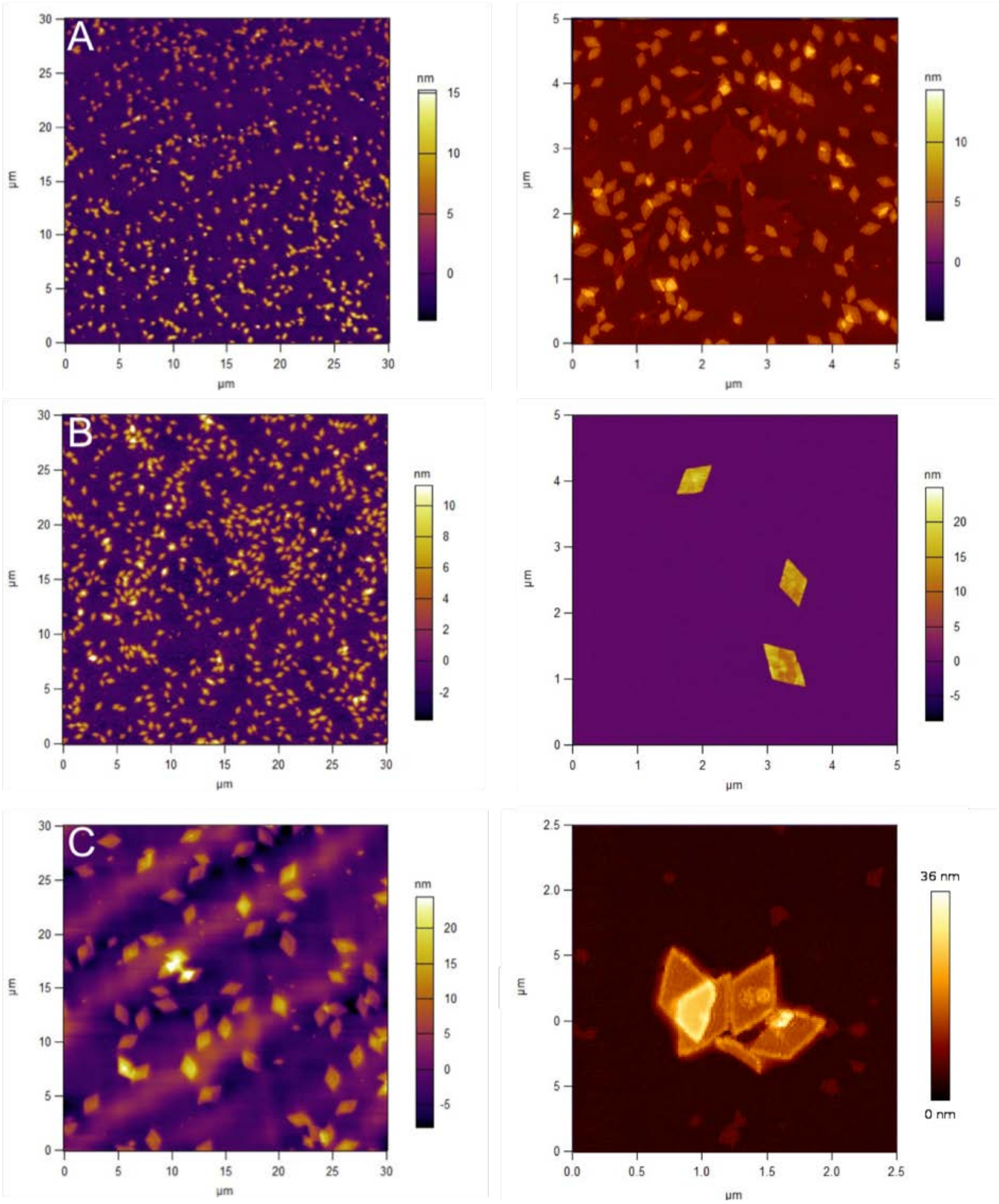

Supplementary Figure 9. Characterisation of PLLA$_{35}$-*b*-PDMAEMA$_{315}$ platelets. AFM images (zoomed-in areas on the right column) of small (A), medium (B), and large (C) PLLA$_{35}$-*b*-PDMAEMA$_{315}$ platelets obtained introducing different amounts of THF in ethanol (0% for A, 6% for B, and 10% for C).

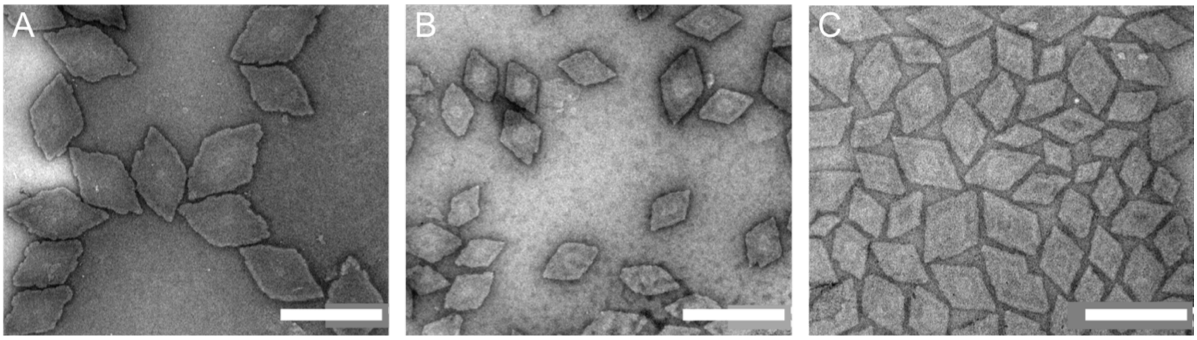

Supplementary Figure 10. Characterisation of $PLLA_{35}$-based platelets. TEM micrographs of $PLLA_{35}$-*b*-$PDMAEMA_{315}$ quaternized (A) and zwitterionic (B) platelets; (C) TEM micrographs of $PLLA_{35}$-*b*-PAA_{400} anionic platelets. Scale bar = 500 nm.

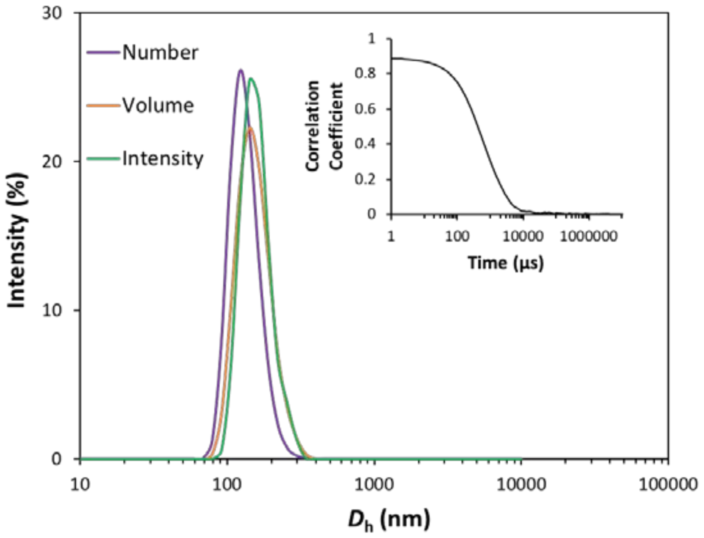

Supplementary Figure 11. DLS analysis of $PLLA_{35}$-*b*-$PDMAEMA_{315}$ spherical micelles (inset: correlation function) measured in water at pH 6.5.

Supplementary Table 4. Quantitative data from DMA adhesion tests using $PLLA_{35}$-based nanopaticles. Data are presented as average ± standard deviation, with , n = 3 and N = 7 for all samples. [a]Small, cationic.

Sample	End to End Adhesive Energy (J/m^2)	Bulk Shear Adhesive Energy (J/m^2)
Control (gel surface)	0.74 ± 0.51	0.81 ± 0.43
Control (homopolymer)	0.91 ± 0.50	0.72 ± 0.30
Spheres	1.03 ± 0.26	1.10 ± 0.71
Platelets[a]	4.94 ± 0.35	9.49 ± 1.61
Cylinders	2.44 ± 0.76	2.20 ± 0.69

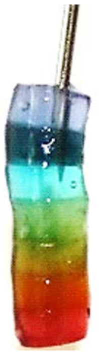

Supplementary Figure 12. Photograph of calcium-alginate hydrogel blocks glued together with $PLLA_{35}$-*b*-$PDMAEMA_{315}$ platelets suspended vertically from a needle.

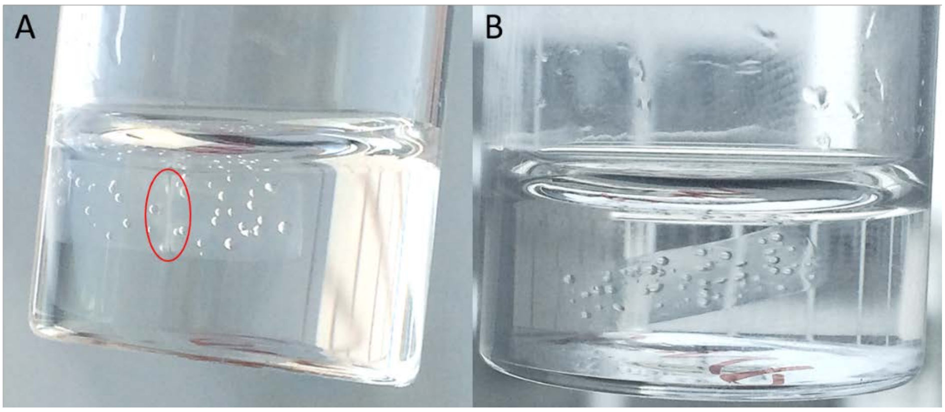

Supplementary Figure 13. Photograph of alginate gel blocks adhered with $PLLA_{35}$-*b*-$PDMAEMA_{315}$ platelets and swelled in water. (A) after 3 h (adhesion joint is circled in red) and (B) after 24 h of swelling.

Supplementary Table 5. Statistical analysis of the bulk shear adhesive energy of alginate hydrogels adhered with $PLLA_{35}$-based nanoparticles of different shapes. Statistical analysis was performed using a two-way ANOVA test, with $p < 0.05$, $n = 3$ and $N = 7$ for all samples.

Sample	Statistically significant?	Adjusted P value
Small *vs.* Medium	****	<0.0001
Small *vs.* Large	****	<0.0001
Medium *vs.* Large	ns	0.1183

Supplementary Table 6. Statistical analysis of the bulk shear adhesive energy of alginate hydrogels adhered with $PLLA_{35}$-*b*-$PDMAEMA_{315}$ platelets with different charges. Statistical analysis was performed using a two-way ANOVA test, with $p < 0.05$, $n = 3$ and $N = 7$ for all samples.

Sample	Statistically significant?	Adjusted P value
Cationic *vs.* Anionic	****	<0.0001
Cationic *vs.* Zwitterionic	****	<0.0001
Cationic *vs.* Neutral	****	<0.0001
Anionic *vs.* Zwitterionic	****	<0.0001
Anionic *vs.* Neutral	****	<0.0001
Zwitterionic *vs.* Neutral	ns	0.9997

Supplementary Table 7. Statistical analysis of the bulk shear adhesive energy of alginate hydrogels adhered with $PLLA_{35}$-based nanoparticles of different shapes. Statistical analysis was performed using a two-way ANOVA test, with $p < 0.05$, $n = 3$ and $N = 7$ for all samples.

Sample	Statistically significant?	Adjusted P value
Control *vs.* Spheres	ns	0.8784
Control *vs.* Cylinders	*	0.0260
Control *vs.* Platelets	****	<0.0001
Spheres *vs.* Cylinders	ns	0.1505
Spheres *vs.* Platelets	****	<0.0001
Cylinders *vs.* Platelets	****	<0.0001

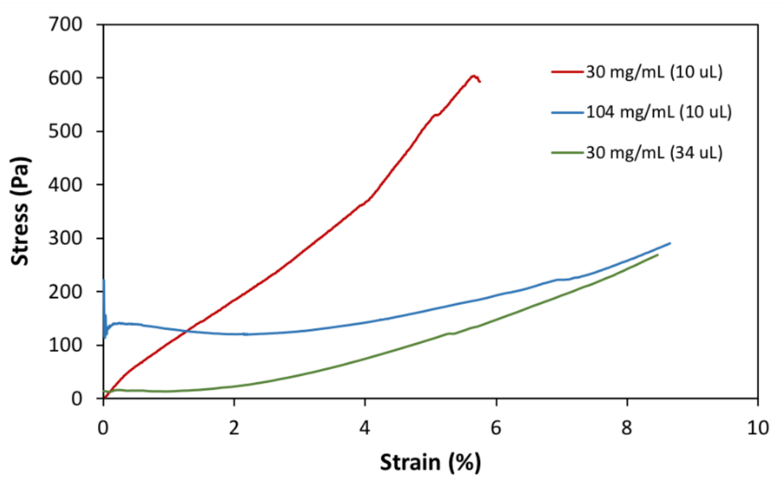

Supplementary Figure 14. Bulk shear stress behavior comparing representative interfacial stresses of different loading of spherical micelles, with a total surface area of 150 cm^2 (red) and 450 cm^2 (blue and green).

Supplementary Table 8. Statistical analysis of the strain at flow point of alginate hydrogels enriched with different concentrations of $PLLA_{35}$-based nanoparticles of different shapes. Statistical analysis was performed using a two-way ANOVA test, with $p < 0.05$ and n = 3 and N = 3 for all samples.

Nanoparticle content (wt%)	Sample	Statistically significant?	Adjusted P value
0.04	Spheres *vs.* Cylinders	**	0.0049
0.04	Spheres *vs.* Platelets	ns	0.1438
0.04	Cylinders *vs.* Platelets	ns	0.3605
0.06	Spheres *vs.* Cylinders	****	<0.0001
0.06	Spheres *vs.* Platelets	**	0.0011
0.06	Cylinders *vs.* Platelets	**	0.0051
0.08	Spheres *vs.* Cylinders	****	<0.0001
0.08	Spheres *vs.* Platelets	****	<0.0001
0.08	Cylinders *vs.* Platelets	****	<0.0001
0.1	Spheres *vs.* Cylinders	****	<0.0001
0.1	Spheres *vs.* Platelets	****	<0.0001
0.1	Cylinders *vs.* Platelets	****	<0.0001
0.12	Spheres *vs.* Cylinders	****	<0.0001
0.12	Spheres *vs.* Platelets	****	<0.0001
0.12	Cylinders *vs.* Platelets	****	<0.0001

Supplementary Table 9. Strain at flow point (SFP) of alginate gels enriched with different concentrations PLLA$_{35}$-*b*-PDMAEMA$_{315}$ platelets of different size. Data are presented as average ± standard deviation, with n = 3 and N = 3 for all samples.

Nanoparticle content (wt%)	SFP (%) Small platelets	SFP (%) Medium platelets	SFP (%) Large platelets
0	12.1 ± 1	12.1 ± 1	12.1 ± 1
0.04	53.2 ± 5.5	33.5 ± 4.8	35.5 ± 4.9
0.06	60.6 ± 5.3	42.1 ± 1.7	40.3 ± 2.6
0.08	64.8 ± 7.1	52.8 ± 4.5	41.2 ± 2.1
0.1	75.7 ± 4.7	60.2 ± 9.4	42.4 ± 1.7
0.12	78.0 ± 5.9	70.0 ± 4.7	51.6 ± 4.7

Supplementary Table 10. Statistical analysis of the strain at flow point of alginate hydrogels enriched with different concentrations of PLLA$_{35}$-*b*-PDMAEMA$_{315}$ platelets of different size. Statistical analysis was performed using a two-way ANOVA test, with $p < 0.05$ and n = 3 and N = 3 for all samples.

Nanoparticle content (wt%)	Sample	Statistically significant?	Adjusted P value
0.04	Small *vs.* Medium	****	<0.0001
0.04	Small *vs.* Large	****	<0.0001
0.04	Medium *vs.* Large	ns	0.8150
0.06	Small *vs.* Medium	****	<0.0001
0.06	Small *vs.* Large	****	<0.0001
0.06	Medium *vs.* Large	ns	0.8472
0.08	Small *vs.* Medium	**	0.0016
0.08	Small *vs.* Large	****	<0.0001
0.08	Medium *vs.* Large	**	0.0024
0.1	Small *vs.* Medium	****	<0.0001
0.1	Small *vs.* Large	****	<0.0001
0.1	Medium *vs.* Large	****	<0.0001
0.12	Small *vs.* Medium	*	0.0464
0.12	Small *vs.* Large	****	<0.0001
0.12	Medium *vs.* Large	****	<0.0001

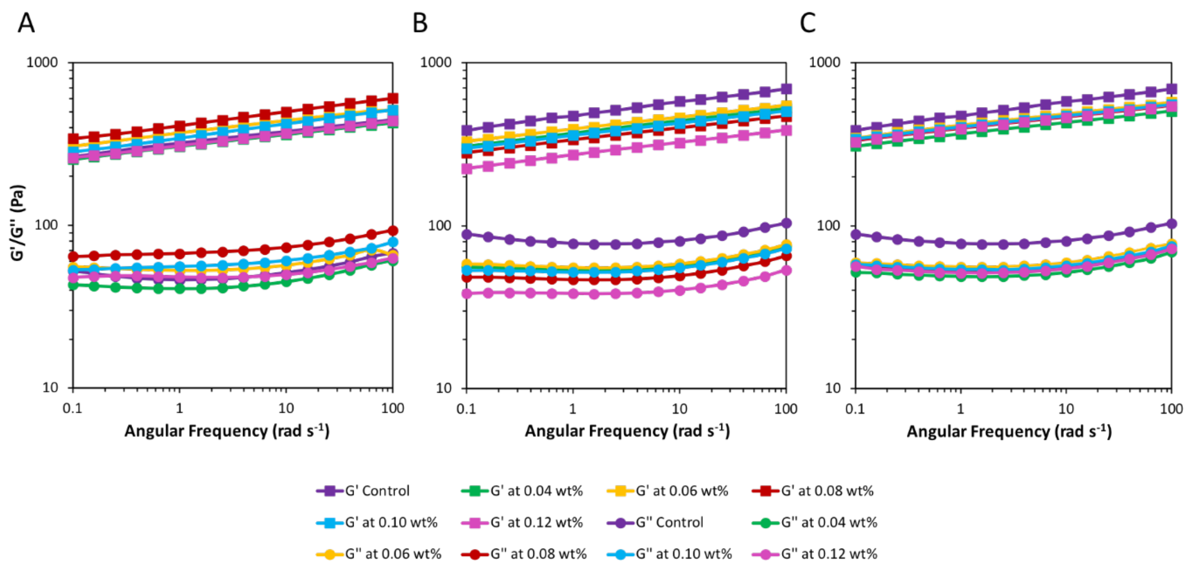

Supplementary Figure 15. Rheological charaterisation of calcium-alginate hydrogels enriched with $PLLA_{35}$-based nanoparticles. Dynamic oscillatory frequency sweeps of alginate hydrogels with various wt% incorporation of $PLLA_{35}$-*b*-$PDMAEMA_{315}$ derived (A) platelets, (B) spherical micelles, and (C) cylindrical micelles.

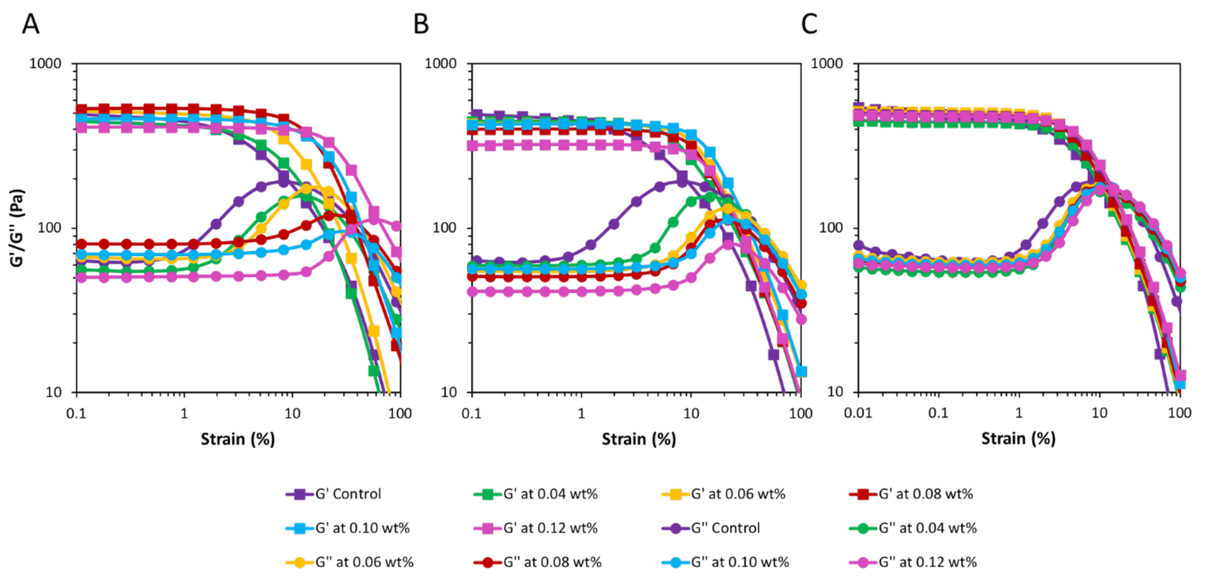

Supplementary Figure 16. Rheological charaterisation of calcium-alginate hydrogels enriched with $PLLA_{35}$-based nanoparticles. Strain-dependent oscillatory rheology (amplitude sweeps) of alginate hydrogels with various wt% incorporation of $PLLA_{35}$-*b*-$PDMAEMA_{315}$ derived (A) platelets, (B) spherical micelles, and (C) cylindrical micelles.

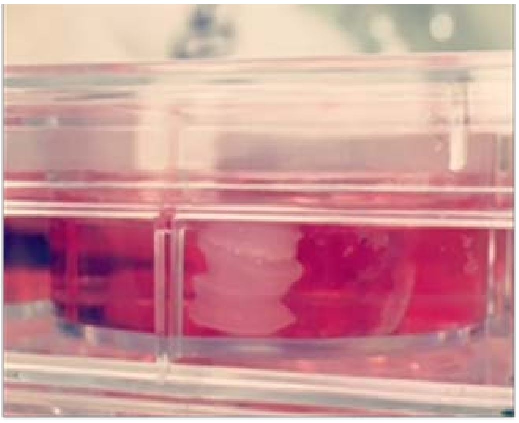

Supplementary Figure 17. Photograph of alginate gel blocks adhered with $PLLA_{35}$-*b*-$PDMAEMA_{315}$ platelets and swelled in cell culture media after 24 h.
