# Supplementary material for: Exploiting the role of nanoparticle shape in enhancing hydrogel adhesive and mechanical properties
Source: Nat Commun. 2020 Mar 17;11:1420. doi: 10.1038/s41467-020-15206-y (PMC7078206; doi:10.1038/s41467-020-15206-y)
Supplement: Supplementary file 3 — Reporting Summary [file 41467_2020_15206_MOESM3_ESM.pdf]

nature research

Corresponding author(s): Andrew Dove

Last updated by author(s): Feb 11, 2020

Reporting Summary

Nature Research wishes to improve the reproducibility of the work that we publish. This form provides structure for consistency and transparency in reporting. For further information on Nature Research policies, see Authors & Referees and the Editorial Policy Checklist .

Statistics

For all statistical analyses, confirm that the following items are present in the figure legend, table legend, main text, or Methods section.

n/a	Confirmed	
☐	☒	The exact sample size (*n*) for each experimental group/condition, given as a discrete number and unit of measurement
☐	☒	A statement on whether measurements were taken from distinct samples or whether the same sample was measured repeatedly
☐	☒	The statistical test(s) used AND whether they are one- or two-sided<br>*Only common tests should be described solely by name; describe more complex techniques in the Methods section.*
☒	☐	A description of all covariates tested
☒	☐	A description of any assumptions or corrections, such as tests of normality and adjustment for multiple comparisons
☐	☒	A full description of the statistical parameters including central tendency (e.g. means) or other basic estimates (e.g. regression coefficient) AND variation (e.g. standard deviation) or associated estimates of uncertainty (e.g. confidence intervals)
☒	☐	For null hypothesis testing, the test statistic (e.g. *F*, *t*, *r*) with confidence intervals, effect sizes, degrees of freedom and *P* value noted<br>*Give P values as exact values whenever suitable.*
☒	☐	For Bayesian analysis, information on the choice of priors and Markov chain Monte Carlo settings
☒	☐	For hierarchical and complex designs, identification of the appropriate level for tests and full reporting of outcomes
☒	☐	Estimates of effect sizes (e.g. Cohen's *d*, Pearson's *r*), indicating how they were calculated

Our web collection on statistics for biologists contains articles on many of the points above.

Software and code

Policy information about availability of computer code

Data collection	RheoCompass ™ software was used for rheology on a Anton Paar Modular Compact Rheometer MCR302; STARe software was used for hydrogel adhesion measurements on a STARe System DMA 1, Mettler Toledo; Wintest software was used for cartilage adhesion measurements on a TA Electroforce 5500.
Data analysis	Statistical analysis has been performed on the adhesion (both of hydrogel and tissue surfaces) and rheology measurements and on the cell viability data using a two-way ANOVA test or a Welch's unpaired t test, with p < 0.05, analysed with GraphPrism 8 software.

For manuscripts utilizing custom algorithms or software that are central to the research but not yet described in published literature, software must be made available to editors/reviewers. We strongly encourage code deposition in a community repository (e.g. GitHub). See the Nature Research guidelines for submitting code & software for further information.

Data

Policy information about availability of data

All manuscripts must include a data availability statement. This statement should provide the following information, where applicable:

- Accession codes, unique identifiers, or web links for publicly available datasets
- A list of figures that have associated raw data
- A description of any restrictions on data availability

The authors declare that the data supporting the findings of this study are available within the paper and its supplementary information file. Raw data underlying the figures presented are available from the corresponding authors upon reasonable request.

Field-specific reporting

Please select the one below that is the best fit for your research. If you are not sure, read the appropriate sections before making your selection.

☒ Life sciences ☐ Behavioural & social sciences ☐ Ecological, evolutionary & environmental sciences

For a reference copy of the document with all sections, see nature.com/documents/nr-reporting-summary-flat.pdf

Life sciences study design

All studies must disclose on these points even when the disclosure is negative.

Sample size	Three human donors were used for the ASC experiment. A minimum of 6 cartilage samples were used for the cartilage adhesion experiments. All cell line experiments were conducted in triplicate. Experimental sample sizes were chosen in order to achieve statistical power and rigourous statistical testing was used to derive significance.
Data exclusions	No data was excluded from analysis.
Replication	All experiments were replicated in line with the number of donors/samples used.
Randomization	Randomization was not relevant as all experiments were conducted using immortalized cell lines or animal tissue obtained from a local abattoir.
Blinding	Blinding was not relevant.

Reporting for specific materials, systems and methods

We require information from authors about some types of materials, experimental systems and methods used in many studies. Here, indicate whether each material, system or method listed is relevant to your study. If you are not sure if a list item applies to your research, read the appropriate section before selecting a response.

Materials & experimental systems

n/a	Involved in the study	
☒	☐	Antibodies
☐	☒	Eukaryotic cell lines
☒	☐	Palaeontology
☒	☐	Animals and other organisms
☒	☐	Human research participants
☒	☐	Clinical data

Methods

n/a	Involved in the study	
☒	☐	ChIP-seq
☒	☐	Flow cytometry
☒	☐	MRI-based neuroimaging

Eukaryotic cell lines

Policy information about cell lines

Cell line source(s)	Human chondrocyte cell line T/C-28a2 was a kind gift from Prof Mary Goldring. Osteoblast cell line SaOS2 was purchased from the European Collection of Authenticated Cell Cultures (ECACC).
Authentication	None of the cell lines were authenticated.
Mycoplasma contamination	None of the cell lines were tested for mycoplasma infection.
Commonly misidentified lines (See ICLAC register)	*Name any commonly misidentified cell lines used in the study and provide a rationale for their use.*
